# A review of mirvetuximab soravtansine-gynx in folate receptor alpha–expressing platinum-resistant ovarian cancer

**DOI:** 10.1093/ajhp/zxaf011

**Published:** 2025-03-24

**Authors:** Judith A Smith, Patrick Medina, Mary Miao, Kathleen N Moore

**Affiliations:** Department of Obstetrics, Gynecology, and Reproductive Sciences, UTHealth Houston McGovern Medical School, Houston, TX; Department of Pharmacy, UTHealth Houston Memorial Hermann Cancer Center, Texas Medical Center, Houston, TX, USA; Department of Medical Affairs, ImmunoGen, Inc., Waltham, MA, USA; Department of Medical Affairs, ImmunoGen, Inc., Waltham, MA, USA; Section of Gynecologic Oncology, Stephenson Cancer Center, University of Oklahoma Health Sciences Center, Oklahoma City, OK; Sarah Cannon Research Institute, Nashville, TN, USA

**Keywords:** antibody-drug conjugate, clinical pharmacology, folate receptor alpha, mirvetuximab soravtansine, ovarian cancer

## Abstract

**Purpose:**

To evaluate the pharmacology, efficacy, safety, and dosing and administration considerations (including adjusted ideal body weight [AIBW] dosing) for mirvetuximab soravtansine-gynx, a first-in-class folate receptor alpha (FRα)–directed antibody-drug conjugate for platinum-resistant ovarian cancer (PROC).

**Summary:**

A literature search was conducted in PubMed using the terms “ovarian cancer” and “mirvetuximab soravtansine” of articles published from inception to April 16, 2024. Relevant publications, abstracts, and clinical trials were reviewed. Mirvetuximab soravtansine-gynx is dosed at 6 mg/kg AIBW every 3 weeks and comprises an FRα-binding antibody, a hydrophilic disulfide linker, and a maytansinoid DM4 payload. Mirvetuximab soravtansine-gynx binds to FRα, which induces receptor-mediated internalization, lysosomal degradation, and release of DM4-containing cytotoxic metabolites. Meaningful anticancer activity in PROC was demonstrated in the single-arm phase 2 SORAYA trial (objective response rate, 32.4%; 95% confidence interval, 23.6%-42.2%) and the confirmatory, randomized phase 3 MIRASOL trial (median progression-free survival with mirvetuximab soravtansine-gynx vs chemotherapy, 5.62 vs 3.98 months; hazard ratio, 0.65; 95% confidence interval, 0.52-0.81; *P* < 0.0001]). Ocular disorders (eg, keratopathy and blurred vision), nausea, diarrhea, and fatigue were among the most common adverse events (AEs) that occurred during clinical trials.

**Conclusion:**

This review of trial data and pharmacology information for AIBW dosing of mirvetuximab soravtansine-gynx will help support its integration into the PROC treatment landscape. The review also discusses recommendations for prophylaxis, monitoring, and management of common AEs, including eye drop regimens, to mitigate ocular events. Mirvetuximab soravtansine-gynx is an effective, novel agent for PROC that targets a newly established biomarker. Established interventions can help mitigate AEs and support the safe use of mirvetuximab soravtansine-gynx.

**Key Points**

- Mirvetuximab soravtansine-gynx, a novel antibody-drug conjugate approved in the US for folate receptor alpha–expressing platinum-resistant ovarian cancer (PROC), is the only agent to demonstrate an overall survival benefit compared to standard-of-care chemotherapy in PROC.
- Mirvetuximab soravtansine-gynx is dosed intravenously at 6 mg/kg adjusted ideal body weight (similar to the imperial-based adjusted body weight) every 3 weeks.
- Recommended prophylaxis, monitoring, and management strategies for common adverse events, such as those used for the mitigation of ocular events, can help ensure continuation of treatment.

Epithelial ovarian cancer (EOC), which encompasses epithelial ovarian, fallopian tube, and primary peritoneal cancer, is the most lethal gynecologic malignancy, with a 5-year relative survival rate of approximately 51%.^1-3^ In 2023, approximately 19,710 new EOC cases occurred in the US, accounting for 1.0% of all new cancer cases.^3^

Standard of care for advanced EOC is a combination of surgery and platinum-based chemotherapy.^2^ Although initial chemotherapy response rates exceed 75%, disease recurs for most patients within 3 years of completing chemotherapy; ultimately, most ovarian cancers develop platinum resistance.^4-6^ Platinum-resistant ovarian cancer (PROC) is defined as disease progression during platinum-based treatment or recurrence within 6 months of the last dose of a platinum-based regimen.^7^ Currently, therapeutic options for PROC are limited, consisting primarily of single-agent nonplatinum chemotherapy with or without bevacizumab, and they have shown considerable toxicity and poor survival outcomes.^8-11^ In the AURELIA trial comparing chemotherapy plus bevacizumab vs chemotherapy alone in patients with PROC, the objective response rate (ORR) was 27.3% with chemotherapy plus bevacizumab, vs 11.8% with chemotherapy alone.^10^ The shortage of biomarker-directed therapies for PROC has also contributed to the challenges of treating individuals living with this malignancy.

Mirvetuximab soravtansine-gynx is a first-in-class antibody-drug conjugate (ADC) that targets folate receptor alpha (FRα) and induces a cytotoxic effect with a tubulin-targeting payload, the maytansinoid DM4.^12,13^ In November 2022, mirvetuximab soravtansine-gynx was granted accelerated approval by the US Food and Drug Administration (FDA) for treatment of adults with FRα-positive PROC who have received 1 to 3 prior systemic treatment regimens, based on findings of meaningful anticancer activity and a safety profile comprising primarily low-grade gastrointestinal, neurosensory, and resolvable ocular adverse events (AEs) in the single-arm SORAYA trial (NCT04296890).^14-16^ Mirvetuximab soravtansine-gynx was subsequently fully approved for this indication based on results from the randomized phase 3 MIRASOL trial (NCT04209855), which demonstrated significant improvements in clinically meaningful endpoints such as progression-free survival (PFS; *P* < 0.0001) and overall survival (OS; *P* = 0.0046) compared to investigator’s choice of chemotherapy, with no new safety signals observed.^17-20^ The combination of mirvetuximab soravtansine-gynx and bevacizumab was also assessed in the phase 1b/2 FORWARD II trial (NCT02606305).^21,22^ For patients with FRα-expressing ovarian, fallopian tube, or primary peritoneal cancer, the National Comprehensive Cancer Network (NCCN) Clinical Practice Guidelines in Oncology (NCCN Guidelines®) recommend mirvetuximab soravtansine-gynx monotherapy as a preferred targeted therapy option in platinum-resistant disease (NCCN category 1).^23^ The NCCN also recommends the combination of mirvetuximab soravtansine-gynx plus bevacizumab as useful in certain circumstances in platinum-resistant disease (NCCN category 2A) and platinum-sensitive disease (NCCN category 2B).^23^

FRα has limited expression on normal tissues but is overexpressed across several tumor types, including ovarian carcinomas, making it an attractive candidate for ADC-directed therapy.^24-26^ Folate receptors facilitate endocytosis of folate, a molecule essential to DNA synthesis, methylation, and repair.^27^ In malignancies, overexpression of FRα may be involved in cancer cell motility, invasion, and proliferation.^28^ Of note, increased FRα expression in ovarian cancer has been associated with worse chemotherapy responses, shorter disease-free intervals, and poorer OS.^29^ FRα expression does not appear to change following chemotherapy, demonstrating the utility of this target in recurrent disease.^30^

ADCs are a relatively new class of targeted drugs; FDA approved the first ADC in 2000 (gemtuzumab ozogamicin for adults with acute myeloid leukemia).^31^ To date, there are 15 approved ADCs worldwide for treatment of hematologic malignancies and solid tumors.^19,31^ Most anticancer ADCs are engineered to eliminate tumor cells while sparing normal tissue; however, this targeted mechanism is greatly affected by the antibody target, linker chemistry, and payload.^31^ The antibody target dictates which tissues are directly affected by the drug’s cytotoxic activity; thus, identifying targets that are not normally expressed on healthy tissue is crucial.^31^ Linker chemistry can affect drug stability and payload release, and payloads can have varying cytotoxic and metabolic characteristics.^31^ Despite similarities in ADC composition across different compounds, each ADC has a distinct pharmacokinetic (PK) profile due to its unique components.^32^ Understanding the PK properties and dosing considerations unique to each ADC therapy is integral to facilitating a treatment plan that supports both patient safety and drug efficacy.

This review discusses important and clinically relevant information for mirvetuximab soravtansine-gynx regarding pharmacology and pharmacokinetics, adjusted ideal body weight (AIBW) dosing, considerations for administration, efficacy, safety, and AE management of this novel, recently approved, biomarker-directed therapy.

## Data sources

An English-based literature search was conducted via PubMed using the terms “mirvetuximab soravtansine” and “ovarian cancer” for articles published between inception and April 16, 2024. ClinicalTrials.gov was also searched using the same terms and dates to locate ongoing mirvetuximab soravtansine-gynx clinical trials. The investigators assessed relevant publicly available articles, abstracts, and regulatory documents identified among citation lists in these sources. In total, 26 mirvetuximab soravtansine-gynx–specific sources were used to support this review.

## Pharmacology of mirvetuximab soravtansine-gynx

**Figure 1.**
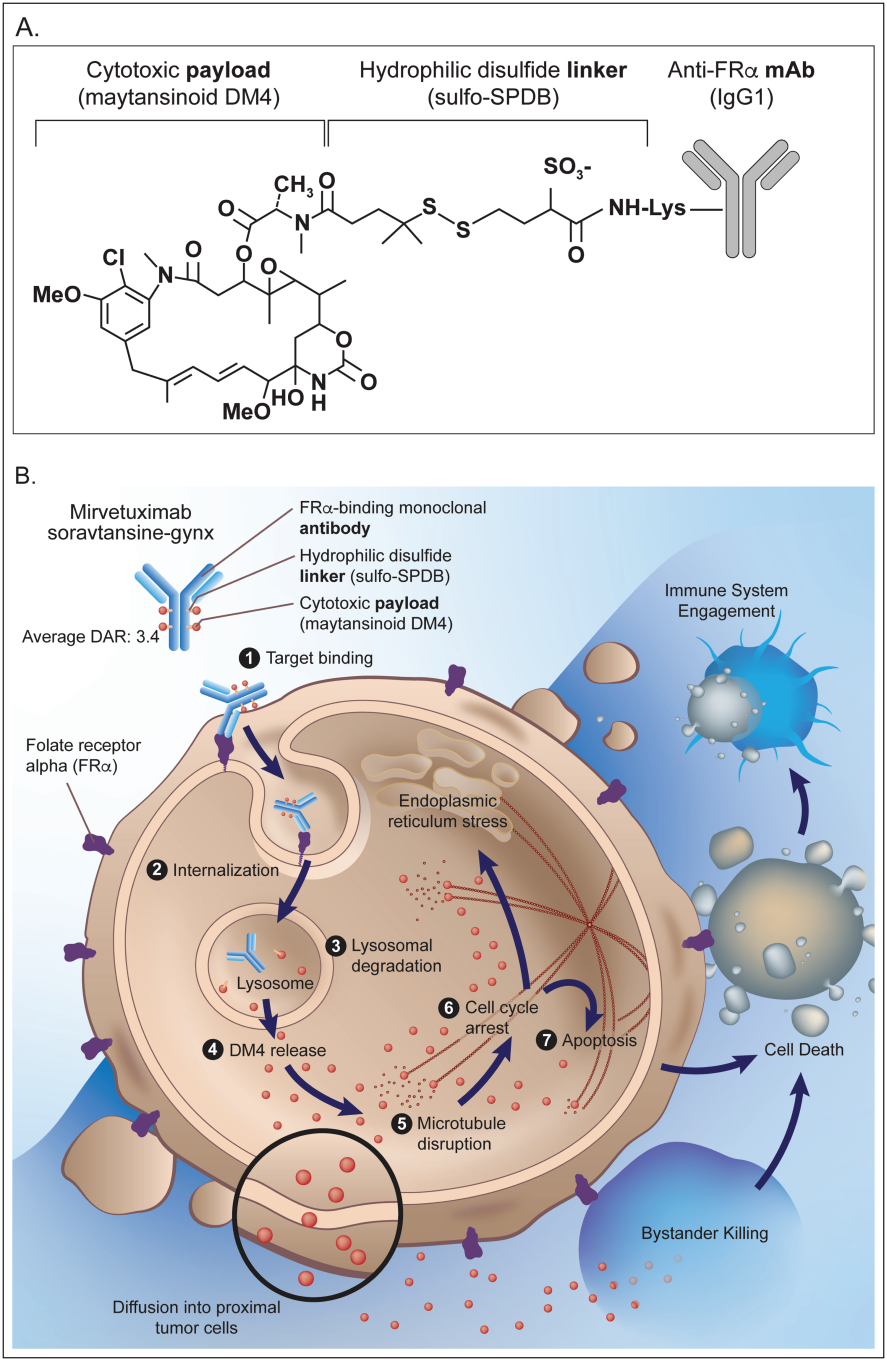
Mirvetuximab soravtansine-gynx structure and proposed mechanism of action. A, Chemical structure of mirvetuximab soravtansine-gynx. Mirvetuximab soravtansine-gynx is composed of a chimeric folate receptor alpha (FRα)–binding immune globulin G1 (IgG1) monoclonal antibody (clone M9346A), a hydrophilic disulfide linker (sulfo-SPDB; 1-(2,5-dioxopyrrolidin-1-yl)oxy-1-oxo-4-(pyridin-2-yl-disulfanyl)butane-2-sulfonic acid), and a cytotoxic maytansine-derived anti-tubulin payload, *N*^2′^-deacetyl-*N*^2′^-(4-mercapto-4-methyl-1-oxopentyl)-maytansine (DM4).^12,19^ The average DM4 (drug) to antibody ratio is 3.4.^19^ B, Mechanism of action of the antibody-drug conjugate mirvetuximab soravtansine-gynx.^19^ 1-4, Upon FRα target binding (1), mirvetuximab soravtansine-gynx undergoes receptor-mediated internalization (2) and lysosomal degradation (3) that results in release of maytansinoid DM4–containing cytotoxic metabolites (eg, *S*-methyl-DM4) (4).^12^ 5-7, Maytansinoids depolymerize tubulin, leading to endoplasmic stress (5),^33^ mitotic arrest (6), and apoptosis (7).^12^ Additionally, disulfide-linked maytansinoid antibody-drug conjugates have demonstrated bystander cytotoxic activity, an effect associated with the formation of active maytansinoid metabolites that diffuse from antigen-targeted cells into neighboring cells.^12^ Finally, immune system engagement may occur following encounters with apoptotic malignant cells.^34^

As illustrated in Figure 1A, mirvetuximab soravtansine-gynx is an ADC composed of an FRα-binding chimeric monoclonal antibody, an *N*-succinimidyl 4-(2-pyridyldithio)-2-sulfobutanoate (sulfo-SPDB) linker, and a maytansinoid DM4 payload.^12,19^ Approximately 72% to 97% of ovarian carcinomas express FRα, with up to 36% of PROC tumors exhibiting high FRα tumor expression (≥75% of tumor cells with an intensity of ≥2+ by immunohistochemistry).^15,24-26^ Of note, studies in preclinical in vitro models have found that tumors with higher FRα expression are more susceptible to the cytotoxic effects of mirvetuximab soravtansine-gynx.^12^ In limited in-human studies, mirvetuximab soravtansine-gynx exposure was not found to significantly impact tumor FRα expression.^26^

Candidates for mirvetuximab soravtansine-gynx must undergo FRα tumor expression testing using the FDA-approved VENTANA FOLR1 (FOLR1-2.1) RxDx assay.^19,35^ Expression of FRα can be evaluated with archival tumor tissues or fresh biopsies.^26^ The positive staining 2+ (PS2+) technique uses immunohistochemistry to evaluate FRα staining intensity and the proportion of tumor cells staining at a specific intensity (eg, 1+, 2+, or 3+).^26,36^ FRα expression level is based on the proportion of viable tumor cells that have greater than or equal to 2+ staining intensity; tumor expression levels are grouped into ranges of 25% to 49% (low), 50% to 74% (medium), or greater than or equal to 75% (high).^26^ The approved indication of mirvetuximab soravtansine-gynx is for patients meeting an FRα clinical expression cutoff of greater than or equal to 75% of viable tumor cells staining with greater than or equal to 2+ intensity (using the FOLR1-2.1 assay).^19,35^ Results of the FOLR1-2.1 diagnostic test must be interpreted by a qualified pathologist.^35^

Mirvetuximab soravtansine-gynx’s mechanism of action requires binding of the anti-FRα antibody to the FRα receptor, which induces receptor-mediated internalization of mirvetuximab soravtansine-gynx, subsequent lysosomal degradation, and release of its payload, maytansinoid DM4–containing cytotoxic metabolites (unconjugated DM4, *S*-methyl-DM4), into the cancer cell (Figure 1B).^12,19^ Maytansinoids bind to a unique β-tubulin site, destabilizing microtubules in a mechanism distinct from that of vinca domain ligands.^37^ After this, the DM4 metabolites induce mitotic arrest and cellular apoptosis.^12^ Disulfide-linked maytansinoid ADCs have been demonstrated to have bystander cytotoxic activity, an effect associated with formation of active maytansinoid metabolites that diffuse from antigen-targeted cells into neighboring cells.^38^ DM4 metabolites may exhibit this bystander effect, thereby enabling mirvetuximab soravtansine-gynx activity against tumors with heterogenous FRα expression.^12^ Each mirvetuximab soravtansine-gynx molecule has an average DM4 (drug) to antibody ratio of 3.4.^19^ Metabolic breakdown of mirvetuximab soravtansine-gynx occurs via the CYP3A4 enzyme (which metabolizes unconjugated DM4 and *S*-methyl-DM4) and proteolysis, which breaks down the antibody portion of mirvetuximab soravtansine-gynx.^19^

## Dose escalation studies of mirvetuximab soravtansine-gynx

Dose escalation studies in the first-in-human phase 1 401 trial (NCT01609556) evaluated mirvetuximab soravtansine-gynx doses ranging from 0.15 to 7 mg/kg total body weight (TBW).^39,40^ The dose-limiting toxicities (DLTs) observed at 5 mg/kg TBW were grade 3 hypophosphatemia and ocular AEs; at 7 mg/kg TBW, grade 3 punctate keratitis occurred.^39^ Additional early analysis of TBW dosing found a trend of dose-dependent ocular symptoms with mirvetuximab soravtansine-gynx exposure.^39^ In an effort to mitigate occurrences of ocular symptoms, the investigators opted to decrease the total mirvetuximab soravtansine-gynx exposure for most patients by using the AIBW,^39^ which is more commonly known as the adjusted body weight (AdjBW) in clinical practice. Two additional dosing cohorts of 7 patients each were added at 5 mg/kg AIBW and 6 mg/kg AIBW.^39^ No DLTs were observed in either group, and no further dose escalation was pursued.^39^ By definition, the maximum tolerated dose was not reached for mirvetuximab soravtansine-gynx based on AIBW dosing once every 3 weeks.^39^ The recommended phase 2 dose for later studies based on these data was 6 mg/kg AIBW once every 3 weeks.^39^

## Pharmacokinetics of mirvetuximab soravtansine-gynx

The PK profile of mirvetuximab soravtansine-gynx is similar to that of other conjugated and nonconjugated therapeutic antibodies.^41^ Following intravenous administration of the first cycle of 6 mg/kg AIBW, mean peak mirvetuximab soravtansine-gynx concentrations occurred near the end of the intravenous infusion.^19^ In contrast, peak concentrations of the unconjugated DM4 payload and its cytotoxic metabolite (*S*-methyl-DM4) were observed on days 2 and 3 after administration, respectively.^19^ Steady-state concentrations of mirvetuximab soravtansine-gynx, DM4, and *S*-methyl-DM4 were reached after one treatment cycle (3 weeks).^19^ Human plasma protein binding of DM4 and *S*-methyl-DM4 was greater than 99% in vitro.^19^*S*-methyl-DM4 and DM4-sulfo-SPDB-lysine were detected in the urine within 24 hours of infusion.^19^ The geometric mean terminal half-life of mirvetuximab soravtansine-gynx, DM4, and *S*-methyl-DM4 after one dose was 4.8 days, 2.8 days, and 5.0 days, respectively.^19,41^

Evaluation of patients with hepatic impairment found no clinically significant differences in PK parameters with mild hepatic impairment (total bilirubin concentration of less than or equal to the upper limit of normal [ULN] and any aspartate aminotransferase [AST] level greater than the ULN or total bilirubin concentration of >1 to 1.5 times the ULN and any AST level) or mild-to-moderate renal impairment (creatinine clearance [CrCl] of 30-89 mL/min).^19,41^ Of note, further studies are needed to evaluate mirvetuximab soravtansine-gynx’s PK parameters among patients with moderate-to-severe hepatic impairment (total bilirubin concentration of >1.5 times the ULN with any AST level) or severe renal impairment (CrCl <30 mL/min).^19^

The PK properties of mirvetuximab soravtansine-gynx were further explored with a population PK model using data from 3 mirvetuximab soravtansine-gynx clinical trials (401, FORWARD I, and SORAYA) to determine the impact of patient demographics and clinical characteristics on mirvetuximab soravtansine-gynx exposure.^42^ This population PK model found that patient AIBW (range, 42.8-96.7 kg), age (range, 34-89 years), and serum albumin concentration (range, 2.0-5.3 g/dL) were statistically significant covariates of specific PK parameters at varying dose levels; however, these factors had a negligible clinical impact following 6 mg/kg AIBW dosing.^42^ Therefore, this analysis further supported the rationale for the selected dose level of 6 mg/kg AIBW.^42^

The DM4 payload is a CYP3A4 substrate; thus, concomitant use of mirvetuximab soravtansine-gynx and strong CYP3A4 inhibitors has the potential to increase exposure to unconjugated DM4.^19^ Although drug-drug interactions have not been thoroughly evaluated during clinical trials or real-world use of mirvetuximab soravtansine-gynx, the population PK model observed no clinically significant interactions.^19,42^ Of note, there has been no evaluation of relevant CYP3A4 inducers to date.

## Mirvetuximab soravtansine-gynx AIBW dosing and administration considerations

Mirvetuximab soravtansine-gynx is dosed at 6 mg/kg AIBW every 3 weeks as an intravenous infusion until disease progression or unacceptable toxicity.^19^

**Figure 2.**
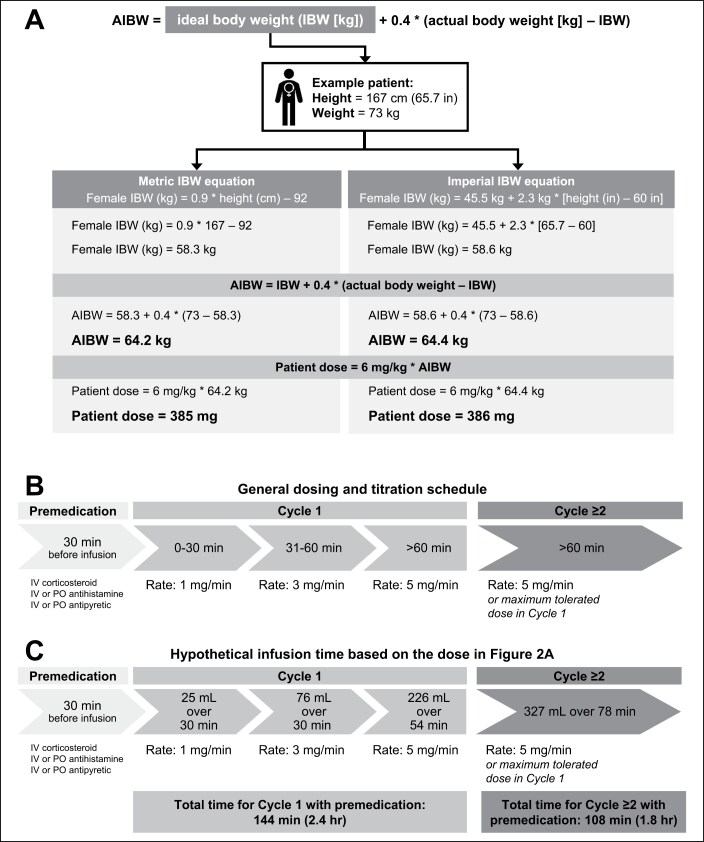
Mirvetuximab soravtansine-gynx adjusted ideal body weight (AIBW) dosing equation and titration schedule.^19,43-46^ A, The metric IBW equation used for mirvetuximab soravtansine-gynx AIBW dosing compared to the imperial IBW equation. The metric-based female IBW equation used to calculate mirvetuximab soravtansine-gynx AIBW dosing can be considered nearly equivalent to the imperial IBW equation for patients who are at least 60 inches tall and weigh more than 45.5 kg. As shown above, under these conditions, the calculated dose for both the metric IBW and imperial IBW equations results in the same volume of drug needed for the dose after rounding. For patients less than 60 inches tall and who weigh less than 45.5 kg, only the metric-based equation should be used. According to the US prescribing information, the calculated dose of 385 mg would require administration of 4 vials (each 20-mL vial contains 5 mg/mL). The total mirvetuximab soravtansine-gynx dose and total bag volume should result in a drug concentration within the range of 1 to 2 mg/mL. For the 385-mg dose with 4 vials, 77 mL of mirvetuximab soravtansine-gynx plus the required 250 mL of 5% dextrose would result in a total volume of 327 mL at a final drug concentration of 1.18 mg/mL. Clinicians should refer to the most recent US prescribing information for best dosing practices. B, General mirvetuximab soravtansine-gynx titration schedule. Mirvetuximab soravtansine-gynx should be titrated to the full rate of infusion during the first cycle if well tolerated. Subsequent cycles are started at the highest tolerated rate. C, Mirvetuximab soravtansine-gynx titration schedule for the hypothetical patient (height, 167 cm; weight, 73 kg) described in A (385 mg mirvetuximab soravtansine-gynx; total volume of 327 mL at a final drug concentration of 1.18 mg/mL). Premedications are administered 30 minutes before infusion. In this hypothetical example, the patient starts with 25 mL of active drug over 30 minutes at a rate of 1 mg/min. If this is well tolerated, the dose is titrated up to administer 76 mL over 30 minutes at a rate of 3 mg/min. If the 3 mg/min rate is well tolerated, the dose is titrated up to 226 mL over 54 minutes, at a rate of 5 mg/min. The total time for cycle 1 (including the premedication period) in this example is 144 minutes (2.4 hours). For cycle 2 and all subsequent cycles, the patient would receive 327 mL over 78 minutes at a rate of 5 mg/min; the total time (including the premedication period) would be 108 minutes (1.8 hours). PO indicates oral; IV, intravenous.

The more commonly recognized term associated with the mirvetuximab soravtansine-gynx AIBW equation is AdjBW; the primary difference is that the IBW is based on the imperial equation rather than the metric IBW equation. Of note, the metric-based equation for female IBW used for mirvetuximab soravtansine-gynx AIBW dosing can be considered equivalent to the imperial IBW equation (45.5 kg + 2.3 kg × [height (in) *–* 60 in]) when patients are at least 60 inches tall and weigh more than 45.5 kg.^43-46^ In the single-arm SORAYA trial that supported accelerated approval, all patients were dosed using 6 mg/kg AIBW every 3 weeks, regardless of their height (range, 146-182 cm) or weight (range, 41.8-124 kg).^15,41^ For patients of shorter height and lower weight, only the metric-based equation should be used. Figure 2A shows the similarities between the metric IBW equation used for mirvetuximab soravtansine-gynx AIBW dosing and the imperial IBW equation.

Importantly, mirvetuximab soravtansine-gynx should be administered using the proper titration schedule (portrayed in Figure 2B) according to the US prescribing information (USPI).^19,47^Figure 2C shows a timeline for a hypothetical infusion. Another important consideration for mirvetuximab soravtansine-gynx administration is use of the correct diluent; mirvetuximab soravtansine-gynx must be diluted with 5% dextrose injection to support drug stability and patient safety.^19,47^ Mirvetuximab soravtansine-gynx should not be mixed with any other drugs or with incompatible diluents, such as 0.9% sodium chloride and lactated Ringer’s solution.^19,47^ Refer to the most current USPI for additional information on mirvetuximab soravtansine-gynx AIBW dosing and administration.^19^

For patients requiring dose reductions for adverse reactions, mirvetuximab soravtansine-gynx should be reduced from 6 mg/kg AIBW to 5 mg/kg AIBW; after this, an additional reduction can be made to 4 mg/kg AIBW.^19^ Patients who cannot tolerate 4 mg/kg AIBW should permanently discontinue mirvetuximab soravtansine-gynx.^19^ Adverse reactions requiring dose modification include keratopathy, uveitis, pneumonitis, peripheral neuropathy, infusion-related reactions (IRRs), and hematologic events.^19^ Key information regarding the occurrence of these adverse events during mirvetuximab soravtansine-gynx clinical trials and measures to mitigate and manage these events is provided later in this review. A summary of dose modifications based on adverse reaction severity, as reported in the USPI, is provided in Table 1.^19^

**Table 1.** Dose Modifications Based on Adverse Reactions (USPI)^19^

Adverse reaction	Severity of adverse reaction^a^	Dosage modification
Keratitis/keratopathy	Nonconfluent superficial keratitis	Monitor
	Confluent superficial keratitis, a cornea epithelial defect, or 3-line or more loss in BCVA	Withhold dose until improved or resolved, then maintain at same dose level or consider dose reduction
	Corneal ulcer or stromal opacity or BCVA 20/200 or worse	Withhold dose until improved or resolved, then reduce by 1 dose level
	Corneal perforation	Permanently discontinue
Uveitis	Grade 1/rare cell in anterior chamber	Monitor
	Grade 2/1-2+ cell or flare in anterior chamber	Withhold dose until grade 1 or less, then maintain dose at same dose level
	Grade 3/3+ cell or flare in anterior chamber	Withhold dose until grade 1 or less, then reduce dose by 1 dose level
	Grade 4/hypopyon	Permanently discontinue
Pneumonitis	Grade 1	Monitor
	Grade 2	Withhold dose until grade 1 or less, then maintain at same dose level or consider dose reduction
	Grade 3 or 4	Permanently discontinue
Peripheral neuropathy	Grade 2	Withhold dose until grade 1 or less, then reduce by 1 dose level
	Grade 3 or 4	Permanently discontinue
IRRs/hypersensitivity	Grade 1	Maintain infusion rate
	Grade 2	Interrupt infusion and administer supportive treatment
		After recovery from symptoms, resume the infusion at 50% of the previous rate and, if no further symptoms appear, increase rate as appropriate until infusion is completed
		Administer additional premedication for future cycles
	Grade 3 or 4	Immediately stop infusion and administer supportive treatment
		Advise patient to seek emergency treatment and immediately notify their healthcare provider if the infusion-related symptoms recur
		Permanently discontinue
Hematologic	Grade 3 or 4	Withhold dose until grade 1 or less, then resume at 1 lower dose level
Other adverse reactions	Grade 3	Withhold dose until grade 1 or less, then resume at 1 lower dose level
	Grade 4	Permanently discontinue

Abbreviations: BCVA, best corrected visual acuity; IRRs, infusion-related reactions; USPI, US Prescribing Information.

^a^Based on National Cancer Institute Common Terminology Criteria for Adverse Events, version 5.0.

## Efficacy of mirvetuximab soravtansine-gynx during clinical trials

### Single-agent mirvetuximab soravtansine-gynx.

Key efficacy results from mirvetuximab soravtansine-gynx clinical trials, along with relevant eligibility criteria and patient demographics, are summarized in Table 2.^15–18,20,21,48–55^

**Table 2.** Completed Clinical Trials of Mirvetuximab Soravtansine-gynx

Trial name and NCT ID	Design and population	Arms	mPFS (95% CI)	ORR (95% CI)	mOS (95% CI)^a^	mDOR (95% CI)	CA-125 (95% CI)^b^
**Single-agent mirvetuximab soravtansine-gynx**							
FORWARD I,^^48,49^^ NCT02631876	Phase 3, open-label, global, randomized trial in PROC (1 to 3 prior lines of therapy) with ≥50% FRα expression (10× staining)	MIRV ITT (n = 248) vs IC chemo ITT (n = 118)	4.1 mo^c^ (NR) vs 4.4 mo^c^ (NR); *P* = 0.897 (NS); HR (95% CI) = 0.98 (0.73-1.31)	22% (NR) vs 12% (NR); *P* = 0.015 (NS); HR (95% CI) = NR	NR^d^; *P* = 0.276 (NS); HR (95% CI) = 0.85 (0.64-1.13)	5.7 mo (NR) vs 7.3 mo (NR); *P* = 0.974; HR (95% CI) = 0.982 (NR)	51% (NR) vs 27% (NR); *P* < 0.001 (NS); HR (95% CI) = NR
		MIRV FRα high (n = 147) vs IC chemo FRα high (n = 71)	4.8 mo (NR) vs 3.3 mo (NR); *P* = 0.049 (NS); HR (95% CI) = 0.69 (0.48-1.00)	24% (NR) vs 10% (NR); *P* = 0.014 (NS); HR (95% CI) = NR	17.3 mo (NR) vs 12.0 mo (NR); *P* = 0.063 (NS); HR (95% CI) = 0.71 (0.49-1.02)	5.7 mo (NR) vs 4.2 mo (NR); *P* = 0.374 (NS); HR (95% CI) = 0.598 (NR)	53% (NR) vs 25% (NR); *P* = 0.001 (NS); HR (95% CI) = NR
SORAYA,^15,16,50^ NCT04296890	Phase 2, single-arm, global trial in PROC (1 to 3 prior lines, including BEV) with high FRα expression^e^	MIRV (n = 106)	4.3 mo (3.7-5.2 mo)	32%^c^ (23.6%-42.2%)	15.0 mo (11.5-18.7 mo)	6.9 mo (5.6-9.7 mo)	47% (35.7%-57.6%)
MIRASOL,^17,18,20^ NCT04209855	Phase 3, open-label, global, randomized trial in PROC (1 to 3 prior lines) with high FRα expression^e^	MIRV (n = 227) vs IC chemo (n = 226)	5.6 mo^c^ (4.3-6.0 mo) vs 4.0 mo^c^ (2.9-4.5 mo); *P* < 0.0001; HR (95% CI) = 0.65 (0.52-0.81)	42% (35.8%-49.0%) vs 16% (11.4%-21.4%); *P* < 0.0001; OR (95% CI) = 3.81 (2.44-5.94)	16.5 mo (14.5-24.6 mo) vs 12.8 mo (10.9-14.4 mo); *P* = 0.0046; HR (95% CI) = 0.67 (0.50-0.89)	6.8 mo (5.6-8.3 mo) vs 4.5 mo (4.2-5.8 mo); *P* = NR; HR (95% CI) = 0.62 (0.40-0.97)	58% (50.5%-65.3%) vs 30% (23.2%-38.2%); *P* = NR; HR (95% CI) = NR
**Mirvetuximab soravtansine-gynx combination therapy**							
FORWARD II,^21,22,51,52^ NCT02606305	Phase 1b/2, multi-arm, global, randomized trial in PROC (1 to 3 prior lines [prior BEV and/or PARPi permitted]) with low, medium, or high FRα expression^e^	MIRV + BEV (n = 94)	8.2 mo (6.8-10.0 mo)	44%^c^ (33%-54%)	NR	9.7 mo (6.9-14.1 mo)	NR
	Phase 1b/2, multi-arm, global, randomized trial in PSOC (1 to 3 prior lines [prior BEV and/or PARPi permitted]) with low, medium, or high FRα expression^e^	MIRV + BEV (n = 31)	9.6 mo (5.4-14.1 mo)	48%^c^ (30%-67%)	NR	12.7 mo (5.0-14.5 mo)	NR
	Phase 1b/2, multi-arm, global, randomized trial in PSOC (≥1 prior line) with low, medium, or high FRα expression^e^	MIRV + carboplatin (n = 18)	15.0 mo (9.9 mo-not reached)	71%^c^ (44%-90%)	NR	Not reached at time of publication (5.7 mo-not reached)	NR
	Phase 1b/2, multi-arm, global, randomized trial in PSOC (1-2 prior lines) with medium or high FRα expression^e^	MIRV + BEV + carboplatin (n = 41)	13.5 mo (9.9-16.3 mo)	83%^c^ (68%-93%)	NR	10.9 mo (8.7-15.2 mo)	NR

Abbreviations: BEV, bevacizumab; CA-125, cancer antigen 125; CI, confidence interval; FRα, folate receptor alpha; HR, hazard ratio; IC chemo, investigator’s choice of chemotherapy; ITT, intent to treat; mDOR, median duration of response; MIRV, mirvetuximab soravtansine-gynx; mOS, median overall survival; mPFS, median progression-free survival; NCT ID, National Clinical Trial identifier; NR, not reported; NS, not significant; OR, odds ratio; ORR, objective response rate; PARPi, poly(adenosine diphosphate [ADP]-ribose) polymerase inhibitors; PROC, platinum-resistant ovarian cancer; PS2+, positive staining 2+; PSOC, platinum-sensitive ovarian cancer.

^a^As applicable, final mOS is reported.

^b^CA-125 response rate according to Gynecologic Cancer InterGroup criteria in evaluable patients.

^c^Primary endpoint.

^d^mOS values at final analysis were not reported in the ITT population. At the time of data cutoff for the primary analysis, mOS was 16.4 months with mirvetuximab soravtansine-gynx vs 14.0 months with investigator’s choice of chemotherapy (HR, 0.82; 95% CI, 0.58-1.15; *P* = 0.248) in the ITT population.

^e^Defined by PS2+ scoring.

Mirvetuximab soravtansine-gynx monotherapy was investigated in the phase 3 FORWARD I trial (NCT02631876) compared to investigator’s choice of chemotherapy in patients with PROC with greater than or equal to 50% tumor FRα expression as defined by a technique that differed from the FDA-approved PS2+ scoring method now used.^35,48,49^ The primary endpoint of PFS was not met; however, exploratory analyses in which FRα levels were rescored using the PS2+ technique found that 34% of patients in the trial had 50% FRα expression levels or lower that should have precluded trial enrollment.^48^ Efficacy data from the FORWARD I trial showed the strongest antitumor activity among patients with at least 75% FRα tumor expression, which was then selected as the cutoff for future mirvetuximab soravtansine-gynx monotherapy trials.^48^ All subsequent mirvetuximab soravtansine-gynx trials, including those reported below, have used the PS2+ scoring method.

The single-arm phase 2 SORAYA trial investigated mirvetuximab soravtansine-gynx monotherapy among patients with advanced, high-grade serous PROC whose tumors expressed greater than or equal to 2+ FRα staining intensity in greater than or equal to 75% of tumor cells (ie, in the “high” group).^15^ Among 105 efficacy-evaluable patients, the ORR was 32.4% (95% confidence interval [CI], 23.6%-42.2%).^15^

The confirmatory phase 3 MIRASOL trial evaluated mirvetuximab soravtansine-gynx monotherapy compared to investigator’s choice of chemotherapy in patients with PROC with the same criterion for FRα tumor expression used in the SORAYA trial.^18^ The primary endpoint of PFS was met, with a median PFS of 5.62 months (95% CI, 4.34-5.95 months) in the mirvetuximab soravtansine-gynx arm compared to 3.98 months (95% CI, 2.86-4.47 months) in the investigator’s choice of chemotherapy arm (hazard ratio [HR], 0.65; 95% CI, 0.52-0.81; *P* < 0.0001).^17,18^ Statistically significant improvements in the key secondary endpoints of ORR and OS were also demonstrated (Table 2).^18^

### Combination therapy.

The FORWARD II phase 1b/2 trial investigated mirvetuximab soravtansine-gynx combination therapy with bevacizumab, carboplatin, pegylated liposomal doxorubicin, bevacizumab and carboplatin, or pembrolizumab in adults with EOC with low, medium, or high (≥25%) FRα expression as defined by the PS2+ scoring method.^21,56^ Therapy with mirvetuximab soravtansine-gynx (6 mg/kg AIBW) and bevacizumab (15 mg/kg based on TBW) every 3 weeks in patients with PROC (n = 94) yielded an ORR of 44% (95% CI, 33%-54%).^21^ Mirvetuximab soravtansine-gynx and bevacizumab combination therapy was also investigated in patients with platinum-sensitive ovarian cancer (PSOC; n = 31); the combination demonstrated an ORR of 48% (95% CI, 30%-67%).^21^ Additionally, FORWARD II evaluated treatment with mirvetuximab soravtansine-gynx (5 or 6 mg/kg AIBW) and carboplatin (ranging from AUC4 to AUC5) every 3 weeks in patients with PSOC (n = 17), which resulted in an ORR of 71% (95% CI, 44%-90%).^51^ Of note, among the patient cohort that received mirvetuximab soravtansine-gynx (6 mg/kg AIBW) plus carboplatin (AUC5) every 3 weeks (n = 9), the ORR was 89% (95% CI, 52%-100%).^56^ Lastly, in another arm of the FORWARD II trial, the mirvetuximab soravtansine-gynx (6 mg/kg AIBW) plus bevacizumab (15 mg/kg based on TBW) plus carboplatin (AUC5) triplet regimen given once every 3 weeks yielded an ORR of 83% (95% CI, 68%-93%) in patients with PSOC (n = 41).^52^

## Overall mirvetuximab soravtansine-gynx safety profile in clinical trials

Ocular AEs (consisting primarily of keratopathy and blurred vision), nausea, diarrhea, abdominal pain, and fatigue were among the most common mirvetuximab soravtansine-gynx–associated AEs in clinical trials.^13,18,19,21,48,51^ Among 682 patients with EOC treated with mirvetuximab soravtansine-gynx monotherapy across 4 clinical trials, peripheral neuropathy occurred in 36% of patients; 3% of patients experienced grade 3 or greater peripheral neuropathy.^19^ Of note, patients with grade 1 or greater baseline peripheral neuropathy, a common AE of microtubule agents, were excluded from these trials.^15^ Other AEs, such as IRRs, pneumonitis, thrombocytopenia, anemia, and alopecia occurred in less than 10% of patients treated with mirvetuximab soravtansine-gynx.^15^ IRRs occurred at similar rates during the SORAYA (9%) and MIRASOL (8%) trials; IRRs during the FORWARD I trial occurred in less than or equal to 15% of participants (specific rate not reported).^19,48^ Of note, in a case study of one SORAYA trial participant with a history of severe anaphylaxis to paclitaxel, mirvetuximab soravtansine-gynx treatment was well tolerated.^57^

As directly compared to single-agent chemotherapy (paclitaxel, pegylated liposomal doxorubicin, and topotecan) in FORWARD I and MIRASOL, mirvetuximab soravtansine-gynx has a differentiated safety profile lacking substantial hematologic toxicity and chemotherapy-induced alopecia.^17,48^ Among patients receiving mirvetuximab soravtansine-gynx in the SORAYA (n = 106) and MIRASOL (n = 218) trials, dose delays occurred in 39% and 54%, dose reductions occurred in 20% and 34%, and permanent discontinuation occurred in 11% and 9% of patients, respectively.^19^

## Ocular events associated with mirvetuximab soravtansine-gynx during clinical trials

Similar to some other ADCs, mirvetuximab soravtansine-gynx is associated with ocular events, which are a relatively new concern for patients and providers treating PROC.^58^ Mirvetuximab soravtansine-gynx treatment should be administered carefully, taking into consideration the black box warning regarding ocular toxicities, which can be found in the USPI.^19^ Mirvetuximab soravtansine-gynx can cause visual impairment, keratopathy, dry eye, photophobia, eye pain, and uveitis.^19^ Among patients with EOC who received mirvetuximab soravtansine-gynx across clinical trials, 59% experienced ocular adverse reactions; the most common (≥5%) reactions were blurred vision (48%), keratopathy (36%), dry eye (27%), cataract (16%), photophobia (14%), and eye pain (10%). Seventy-five patients (11%) experienced a grade 3 ocular event, and 2 patients (0.3%) experienced a grade 4 event (keratopathy and cataract). Median time to onset for the first ocular adverse reaction was 5.1 weeks (range, 0.1-68.6 weeks). Of the patients who experienced ocular events, 53% had complete resolution and 38% had partial improvement (defined as a decrease in severity by one or more grades from the worst grade) at last follow-up. Ocular adverse reactions led to permanent discontinuation of mirvetuximab soravtansine-gynx in 1% of patients.^19^

## Mitigation and management of AEs associated with mirvetuximab soravtansine-gynx

### Ocular.

The mitigation and management of ocular events associated with mirvetuximab soravtansine-gynx have been expanded upon and published previously in a review.^59^ All patients initiating mirvetuximab soravtansine-gynx must undergo ophthalmic examinations, including visual acuity evaluation and slit lamp examination, before initiating mirvetuximab soravtansine-gynx and every other cycle through the first 8 cycles, and then as clinically indicated. Patients must also be instructed to use ophthalmic topical steroids and lubricating eye drops.^19,59^ Patients should administer 1 drop of ophthalmic topical steroids (eg, 1% prednisolone acetate ophthalmic suspension) in each eye 6 times daily starting the day before each infusion until day 4; then, they should administer 1 drop in each eye 4 times daily for days 5 through 8 of each mirvetuximab soravtansine-gynx cycle.^19,59^ Lubricating eye drops should be used liberally (≥4 times daily) during treatment with mirvetuximab soravtansine-gynx (preservative-free solutions are recommended).^19^ Clinicians should instruct patients to administer lubricating eye drops at least 10 minutes after steroid eye drops.^19^ Additionally, patients should be advised to avoid wearing contact lenses unless directed by their eye care professional (eg, optometrist or ophthalmologist) and take other ocular precautions, such as cleaning eyelid margins, applying warm compresses, and wearing sunglasses in daylight. ^59^ Proactive monitoring for ocular symptoms, including vision changes, and swift referrals of patients to an eye care professional are vital to ocular AE mitigation and management.^59^ Future recommendations may suggest the use of ophthalmic topical steroids only if patients are experiencing keratopathy or ocular issues that present concern for cataract development, rather than for all patients.

### Gastrointestinal.

Before each mirvetuximab soravtansine-gynx infusion and thereafter as needed, clinicians should administer an oral or intravenous antiemetic, such as a serotonin receptor antagonist (eg, granisetron, ondansetron, or palonosetron) with dexamethasone.^15,19^ Mirvetuximab soravtansine-gynx should be considered to have moderate emetogenicity.^60^ Incidence of diarrhea after infusion, if experienced, can be managed with antidiarrheals (eg, loperamide) at the discretion of the clinician.^15^ There is currently no formal guidance for treating the abdominal pain that may occur with mirvetuximab soravtansine-gynx treatment.

### IRRs.

Signs and symptoms of IRRs vary widely and may include headache, fever, facial flushing, pruritus, myalgia, nausea, chest tightness, dyspnea, vomiting, erythema, abdominal discomfort, diaphoresis, shivers, light-headedness, hypotension, palpitations, and somnolence.^15^ Because anaphylaxis may occur during an infusion, clinicians should have medications such as corticosteroids, epinephrine, and antihistamines on standby and treat patients per their institutional protocol.^15^ Premedication to prevent IRRs consists of an intravenous corticosteroid (eg, dexamethasone 10 mg), an oral or intravenous antihistamine (eg, diphenhydramine 25 to 50 mg), and an oral or intravenous antipyretic (eg, acetaminophen 325 to 650 mg) given 30 minutes before infusion.^19^ Patients should be informed of the signs and symptoms of IRRs and know to seek medical care if symptoms develop or recur after leaving the clinic.^15^ The infusion can be resumed at the same rate after a grade 1 IRR.^19^ In the case of grade 2 IRRs, resume the infusion at 50% of the previous rate upon symptomatic recovery; if no symptoms recur, the rate can be increased as appropriate until completion.^19^ Clinicians can also consider including additional premedications for these patients in advance of future infusions, as clinically appropriate.^19^ For IRRs of grade 3 or higher, permanently discontinue mirvetuximab soravtansine-gynx.^19^

### Pneumonitis.

Clinicians should monitor patients for signs and symptoms of pneumonitis, including hypoxia, cough, dyspnea, and interstitial infiltrates on radiologic examination.^19^ Pneumonitis is a serious AE that can occur with multiple ADCs^61^; corticosteroids are typically administered to mitigate the symptoms. The management of pneumonitis, however, differs across compounds. For example, patients on mirvetuximab soravtansine-gynx with persistent or recurrent cases of grade 2 pneumonitis should have mirvetuximab soravtansine-gynx held until resolution to a lower grade; then, patients can resume mirvetuximab soravtansine-gynx treatment at the same dose level or a reduced dose at the discretion of their clinician.^19^ Patients with pneumonitis of grade 3 or higher should permanently discontinue mirvetuximab soravtansine-gynx.^19^ Patients should be advised to immediately notify their clinician if they are experiencing shortness of breath, cough, or respiratory distress.^15^

## Relevance to patient care and clinical practice

Mirvetuximab soravtansine-gynx is the first biomarker-directed therapy approved for patients with PROC, a difficult-to-treat population for which an unmet need exists.^62^ Mirvetuximab soravtansine-gynx is an efficacious treatment option with a manageable safety profile for patients with PROC. This review of current clinical trial data provides essential pharmacology information for AIBW dosing of mirvetuximab soravtansine-gynx to help facilitate its implementation in treatment of PROC. Recommended monitoring and prophylactic eye drop regimens to prevent ocular events as well as for management of other common AEs were discussed. Mitigation and management strategies to reduce the incidence and severity of AEs are important to help ensure optimal patient care on mirvetuximab soravtansine-gynx. Ongoing trials investigating both single-agent mirvetuximab soravtansine-gynx and various therapeutic combinations in different treatment settings are outlined in Table 3.^53,54,55,63–65^

**Table 3.** Ongoing Clinical Trials of Mirvetuximab Soravtansine-gynx

Trial name and NCT ID (status)	Design and population	Primary endpoint	Key secondary endpoint	Other secondary endpoints^a^
**Single-agent mirvetuximab soravtansine-gynx**				
PICCOLO,^53,63^ NCT05041257 (enrollment complete)	Phase 2, single-arm, global trial in PSOC (≥2 prior lines) with high FRα expression^b^ of MIRV monotherapy (n = ~75)	ORR (IA)	DOR (IA)	TEAEs; CA-125 response^c^; PFS (IA); OS; ORR, DOR, and PFS by BICR^d^
424,^64^ NCT06365853 (not yet recruiting)	Phase 2, global trial in PROC and PSOC with high FRα expression^b^ of MIRV monotherapy with participants randomized to 1 of 2 ocular AE risk mitigation strategy arms (primary prophylactic steroid eye drops vs primary prophylactic vasoconstricting eye drops)	Number of asymptomatic participants with MIRV-related grade ≥2 corneal AEs	None	Number of participants with all ocular TEAEs using corticosteroid vs vasoconstricting eye drop primary prophylaxis; number of asymptomatic vs symptomatic participants with MIRV-related corneal AEs and all ocular TEAEs; number of participants with MIRV-related corneal AEs and all ocular TEAEs using corticosteroid vs vasoconstricting eye drop primary prophylaxis; NEI VFQ-25 composite score; MIRV AUC; MIRV *C*_max_; MIRV *C*_trough_
**Mirvetuximab soravtansine-gynx combination therapy**				
GLORIOSA,^54,65,66^ NCT05445778 (currently enrolling)	Phase 3, open-label, global, randomized trial in PSOC (4 to 8 prior cycles of platinum-based triplet therapy in 2L without disease progression) with high FRα expression^b^ of MIRV + BEV vs BEV for 2L maintenance (n = ~420)	PFS (IA and BICR^d^)	OS	Safety and tolerability; PFS2; ORR (IA and BICR^d^); DOR (IA and BICR^d^); DFS (IA and BICR^d^); CA-125 response;^c^ PROs
420 trial,^55^ NCT05456685 (currently enrolling)	Phase 2, open-label, global trial in PSOC (1 prior line of platinum-based chemotherapy) with low, medium, or high FRα expression^b^ of MIRV + carboplatin (n = ~110)	ORR (IA and BICR^d^)	ORR (IA and BICR^d^)	DOR (IA and BICR^d^); PFS (IA and BICR^d^); OS; CA-125 response;^c^ TEAEs

Abbreviations: 2L, second line; AE, adverse event; AUC, area under the curve; BEV, bevacizumab; BICR, blinded independent central review; CA-125, cancer antigen 125; *C*_max_, maximum serum concentration; *C*_trough_, trough concentration; DFS, disease-free survival; DOR, duration of response; FRα, folate receptor alpha; IA, investigator assessed; MIRV, mirvetuximab soravtansine-gynx; NCT ID, National Clinical Trial identifier; NEI VFQ-25, National Eye Institute Visual Function Questionnaire 25; ORR, objective response rate; OS, overall survival; PFS, progression-free survival; PFS2, time to second disease progression; PROC, platinum-resistant ovarian cancer; PROs, patient-reported outcomes; PS2+, positive staining 2+; PSOC, platinum-sensitive ovarian cancer; TEAEs, treatment-emergent adverse events.

^a^Other secondary endpoints are described and ordered as listed on ClinicalTrials.gov.

^b^Defined by PS2+ scoring.

^c^CA-125 response rate according to Gynecologic Cancer InterGroup criteria in evaluable patients.

^d^By BICR as a sensitivity analysis.

## Conclusion

Mirvetuximab soravtansine-gynx is a first-in-class FRα-directed ADC that is FDA approved for the treatment of adult patients with FRα-positive PROC who have received 1 to 3 prior systemic treatment regimens.^19^ Dosed at 6 mg/kg AIBW, mirvetuximab soravtansine-gynx is an intravenous infusion given every 3 weeks until disease progression or unacceptable toxicity.^19^ Clinicians must diligently use mitigation strategies in patients, monitor them closely, and collaborate with a multidisciplinary care team to lower the risk and severity of adverse reactions, such as resolvable ocular events, gastrointestinal symptoms, IRRs, and pneumonitis. Recommendations for AIBW dosing, administration, and AE mitigation can support the safe use of mirvetuximab soravtansine-gynx in patients with PROC.

The results observed with mirvetuximab soravtansine-gynx monotherapy in patients with PROC have positioned mirvetuximab soravtansine-gynx as a new standard-of-care treatment in those with FRα-positive platinum-resistant disease.^18^ Additional clinical trials are evaluating mirvetuximab soravtansine-gynx monotherapy and combination therapy in FRα-positive PSOC, including the single-arm phase 2 PICCOLO trial (NCT05041257; mirvetuximab soravtansine-gynx as third-line or greater therapy), the single-arm phase 2 420 trial (NCT05456685; mirvetuximab soravtansine-gynx and carboplatin as second-line therapy), and the randomized phase 3 GLORIOSA trial (NCT05445778; mirvetuximab soravtansine-gynx and bevacizumab in the second line as maintenance therapy).^53–55,63,64^

## Data Availability

No new data were generated or analyzed in support of this work.

## References

[1] Chen LM , Berek JS. Epithelial carcinoma of the ovary, fallopian tube, and peritoneum: clinical features and diagnosis. In: Lexicomp Online [proprietary data]. UpToDate, Inc.; 2022. Accessed May 17, 2022.

[2] Kuroki L , Guntupalli SR. Treatment of epithelial ovarian cancer. BMJ. 2020;371:m3773. 33168565 10.1136/bmj.m3773

[3] Surveillance, Epidemiology, and End Results Program, National Cancer Institute. Cancer stat facts: ovarian cancer. Accessed February 21, 2023. https://seer.cancer.gov/statfacts/html/ovary.html

[4] Chien J , Kuang R, Landen C, et al Platinum-sensitive recurrence in ovarian cancer: the role of tumor microenvironment. Front Oncol. 2013;3:251. 24069583 10.3389/fonc.2013.00251 PMC3781360

[5] Indini A , Nigro O, Lengyel CG, et al Immune-checkpoint inhibitors in platinum-resistant ovarian cancer. Cancers. 2021;13(7):1663. 33916221 10.3390/cancers13071663 PMC8037571

[6] Kemp Z , Ledermann J. Update on first-line treatment of advanced ovarian carcinoma. Int J Womens Health. 2013;5:45-51. 23378788 10.2147/IJWH.S30231 PMC3558307

[7] Wilson MK , Pujade-Lauraine E, Aoki D, et al Fifth Ovarian Cancer Consensus Conference of the Gynecologic Cancer InterGroup: recurrent disease. Ann Oncol. 2017;28(4):727-732. 27993805 10.1093/annonc/mdw663 PMC6246494

[8] Gaillard S , Oaknin A, Ray-Coquard I, et al Lurbinectedin versus pegylated liposomal doxorubicin or topotecan in patients with platinum-resistant ovarian cancer: a multicenter, randomized, controlled, open-label phase 3 study (CORAIL). Gynecol Oncol. 2021;163(2):237-245. 34521554 10.1016/j.ygyno.2021.08.032

[9] Mutch DG , Orlando M, Goss T, et al Randomized phase III trial of gemcitabine compared with pegylated liposomal doxorubicin in patients with platinum-resistant ovarian cancer. J Clin Oncol. 2007;25(19):2811-2818. 17602086 10.1200/JCO.2006.09.6735

[10] Pujade-Lauraine E , Hilpert F, Weber B, et al Bevacizumab combined with chemotherapy for platinum-resistant recurrent ovarian cancer: the AURELIA open-label randomized phase III trial. J Clin Oncol. 2014;32(13):1302-1308. 24637997 10.1200/JCO.2013.51.4489

[11] ten Bokkel Huinink W , Gore M, Carmichael J, et al Topotecan versus paclitaxel for the treatment of recurrent epithelial ovarian cancer. J Clin Oncol. 1997;15(6):2183-2193. 9196130 10.1200/JCO.1997.15.6.2183

[12] Ab O , Whiteman KR, Bartle LM, et al IMGN853, a folate receptor-alpha (FRα)-targeting antibody-drug conjugate, exhibits potent targeted antitumor activity against FRα-expressing tumors. Mol Cancer Ther. 2015;14(7):1605-1613. 25904506 10.1158/1535-7163.MCT-14-1095

[13] Moore KN , Martin LP, O’Malley DM, et al Safety and activity of mirvetuximab soravtansine (IMGN853), a folate receptor alpha-targeting antibody-drug conjugate, in platinum-resistant ovarian, fallopian tube, or primary peritoneal cancer: a phase I expansion study. J Clin Oncol. 2017;35(10):1112-1118. 28029313 10.1200/JCO.2016.69.9538 PMC5559878

[14] Food and Drug Administration. BLA accelerated approval. Accessed February 21, 2023. https://www.accessdata.fda.gov/drugsatfda_docs/appletter/2022/761310Orig1s000ltr.pdf

[15] Matulonis UA , Lorusso D, Oaknin A, et al Efficacy and safety of mirvetuximab soravtansine in patients with platinum-resistant ovarian cancer with high folate receptor alpha expression: results from the SORAYA study. J Clin Oncol. 2023;41(13):2436-2445. 36716407 10.1200/JCO.22.01900 PMC10150846

[16] ImmunoGen, Inc. A study of mirvetuximab soravtansine in platinum-resistant, advanced high-grade epithelial ovarian, primary peritoneal, or fallopian tube cancers with high folate receptor-alpha expression (SORAYA). In: ClinicalTrials.gov. Updated August 7, 2024. Accessed February 10, 2025. https://clinicaltrials.gov/ct2/show/NCT04296890

[17] Moore KN , Angelergues A, Konecny GE, et al Phase III MIRASOL (GOG 3045/ENGOT-ov55) study: mirvetuximab soravtansine vs. investigator’s choice of chemotherapy in platinum-resistant, advanced high-grade epithelial ovarian, primary peritoneal or fallopian tube cancers with high folate receptor-alpha (FRα) expression. Abstract (LBA 5507) presented at: American Society of Clinical Oncology Annual Meeting; June 2023; Chicago, IL.

[18] Moore KN , Angelergues A, Konecny GE, et al Mirvetuximab soravtansine in FRα-positive, platinum-resistant ovarian cancer. N Engl J Med. 2023;389(23):2162-2174. 38055253 10.1056/NEJMoa2309169

[19] Elahere. Package insert. ImmunoGen, Inc.; 2024.

[20] AbbVie. A study of mirvetuximab soravtansine vs. investigator’s choice (IC) of chemotherapy in platinum-resistant, advanced high-grade epithelial ovarian, primary peritoneal, or fallopian tube cancers with high folate receptor-alpha (FRα) expression (MIRASOL). In: ClinicalTrials.gov. Updated January 7, 2025. Accessed February 10, 2025. https://clinicaltrials.gov/ct2/show/NCT04209855

[21] Gilbert L , Oaknin A, Matulonis UA, et al Safety and efficacy of mirvetuximab soravtansine, a folate receptor alpha (FRα)-targeting antibody-drug conjugate (ADC), in combination with bevacizumab in patients with platinum-resistant ovarian cancer. Gynecol Oncol. 2023;170:241-247. 36736157 10.1016/j.ygyno.2023.01.020

[22] ImmunoGen, Inc. Study of mirvetuximab soravtansine in combination with bevacizumab, carboplatin, pegylated liposomal doxorubicin, pembrolizumab, or bevacizumab + carboplatin in participants with folate receptor alpha (FRα) positive advanced epithelial ovarian cancer, primary peritoneal, or fallopian tube cancer. In: ClinicalTrials.gov. Updated December 17, 2021. Accessed February 8, 2023. https://clinicaltrials.gov/ct2/show/NCT02606305

[23] Referenced with permission from the NCCN Clinical Practice Guidelines in Oncology (NCCN Guidelines®) for Ovarian Cancer/Fallopian Tube Cancer/Primary Peritoneal Cancer V.3.2024. © National Comprehensive Cancer Network, Inc. 2024. All rights reserved. Accessed August 1, 2024. To view the most recent and complete version of the guideline, go online to NCCN.org. NCCN makes no warranties of any kind whatsoever regarding their content, use or application and disclaims any responsibility for their application or use in any way.

[24] Markert S , Lassmann S, Gabriel B, et al Alpha-folate receptor expression in epithelial ovarian carcinoma and non-neoplastic ovarian tissue. Anticancer Res. 2008;28(6a):3567-3572. 19189636

[25] Kalli KR , Oberg AL, Keeney GL, et al Folate receptor alpha as a tumor target in epithelial ovarian cancer. Gynecol Oncol. 2008;108(3):619-626. 18222534 10.1016/j.ygyno.2007.11.020 PMC2707764

[26] Martin LP , Konner JA, Moore KN, et al Characterization of folate receptor alpha (FRα) expression in archival tumor and biopsy samples from relapsed epithelial ovarian cancer patients: a phase I expansion study of the FRα-targeting antibody-drug conjugate mirvetuximab soravtansine. Gynecol Oncol. 2017;147(2):402-407. 28843653 10.1016/j.ygyno.2017.08.015 PMC6893864

[27] Ledermann JA , Canevari S, Thigpen T. Targeting the folate receptor: diagnostic and therapeutic approaches to personalize cancer treatments. Ann Oncol. 2015;26(10):2034-2043. 26063635 10.1093/annonc/mdv250

[28] Siu MK , Kong DS, Chan HY, et al Paradoxical impact of two folate receptors, FRα and RFC, in ovarian cancer: effect on cell proliferation, invasion and clinical outcome. PLoS One. 2012;7(11):e47201. 23144806 10.1371/journal.pone.0047201 PMC3492371

[29] Chen YL , Chang MC, Huang CY, et al Serous ovarian carcinoma patients with high alpha-folate receptor had reducing survival and cytotoxic chemo-response. Mol Oncol. 2012;6(3):360-369. 22265591 10.1016/j.molonc.2011.11.010 PMC5528335

[30] Crane LM , Arts HJ, van Oosten M, et al The effect of chemotherapy on expression of folate receptor-alpha in ovarian cancer. Cell Oncol. 2012;35(1):9-18. 10.1007/s13402-011-0052-6 PMC3268989 21647742

[31] Fu Z , Li S, Han S, et al Antibody drug conjugate: the “biological missile” for targeted cancer therapy. Signal Transduct Target Ther. 2022;7(1):93. 35318309 10.1038/s41392-022-00947-7 PMC8941077

[32] Lin K , Tibbitts J, Shen BQ. Pharmacokinetics and ADME characterizations of antibody–drug conjugates. Methods Mol Biol. 2013;1045:117-131. 23913144 10.1007/978-1-62703-541-5_7

[33] Liu GY , Chen SC, Lee GH, et al Precise control of microtubule disassembly in living cells. EMBO J. 2022;41(15):e110472. 35686621 10.15252/embj.2021110472 PMC9340485

[34] Manzano A , Ocaña A. Antibody-drug conjugates: a promising novel therapy for the treatment of ovarian cancer. Cancers. 2020;12(8):2223. 32784819 10.3390/cancers12082223 PMC7464539

[35] Ventana Medical Systems, Inc., and Roche Diagnostics International, Inc. Ventana FOLR1 (FOLR1-2.1) RxDx Assay (Interpretation Guide for EOC). Ventana Medical Systems, Inc., and Roche Diagnostics International, Inc.; 2022.

[36] Moore K , Oza A, Colombo N, et al FORWARD I (GOG 3011): a phase III study of mirvetuximab soravtansine, a folate receptor alpha (FRα)-targeting antibody-drug conjugate (ADC), versus chemotherapy in patients (pts) with platinum-resistant ovarian cancer (PROC). Ann Oncol. 2019;30(suppl 5):V403.

[37] Prota AE , Bargsten K, Diaz JF, et al A new tubulin-binding site and pharmacophore for microtubule-destabilizing anticancer drugs. Proc Natl Acad Sci USA. 2014;111(38):13817-13821. 25114240 10.1073/pnas.1408124111 PMC4183314

[38] Kovtun YV , Audette CA, Ye Y, et al Antibody-drug conjugates designed to eradicate tumors with homogeneous and heterogeneous expression of the target antigen. Cancer Res. 2006;66(6):3214-3221. 16540673 10.1158/0008-5472.CAN-05-3973

[39] Moore KN , Borghaei H, O’Malley DM, et al Phase 1 dose-escalation study of mirvetuximab soravtansine (IMGN853), a folate receptor alpha-targeting antibody-drug conjugate, in patients with solid tumors. Cancer. 2017;123(16):3080-3087. 28440955 10.1002/cncr.30736 PMC6896318

[40] ImmunoGen, Inc. First-in-human study to evaluate the safety, tolerability, pharmacokinetics and pharmacodynamics of mirvetuximab soravtansine in adults with ovarian cancer and other folate receptor 1 (FOLR1)-positive solid tumors (IMGN853-0401). In: ClinicalTrials.gov. Updated February 17, 2021. Accessed February 10, 2025. https://clinicaltrials.gov/ct2/show/NCT01609556

[41] Tu YP , Hanze E, Zhu F, et al Population pharmacokinetics of mirvetuximab soravtansine in patients with folate receptor-alpha positive ovarian cancer: the antibody-drug conjugate, payload and metabolite. Br J Clin Pharmacol. 2024;90(2):568-581. 37872122 10.1111/bcp.15937

[42] Moore KN , Lorusso D, Oaknin A, et al Population pharmacokinetic (PK) analysis of mirvetuximab soravtansine (MIRV) in patients with folate receptor α (FRα)–positive cancer. Abstract (605P) presented at: European Society of Medical Oncology Congress; September 2022; Paris, France.

[43] Barras M , Legg A. Drug dosing in obese adults. Aust Prescr. 2017;40(5):189-193. 29109603 10.18773/austprescr.2017.053 PMC5662437

[44] Peterson CM , Thomas DM, Blackburn GL, et al Universal equation for estimating ideal body weight and body weight at any BMI. Am J Clin Nutr. 2016;103(5):1197-1203. 27030535 10.3945/ajcn.115.121178 PMC4841935

[45] Pai MP , Paloucek FP. The origin of the “ideal” body weight equations. Ann Pharmacother. 2000;34(9):1066-1069. 10981254 10.1345/aph.19381

[46] ClinCalc LLC. Ideal Body Weight Calculator. Accessed August 28, 2023. https://clincalc.com/Kinetics/IdealBW.aspx

[47] Luo S , McSweeney KM, Wang T, et al Defining the right diluent for intravenous infusion of therapeutic antibodies. MAbs. 2020;12(1):1685814. 31774346 10.1080/19420862.2019.1685814 PMC6927757

[48] Moore KN , Oza AM, Colombo N, et al Phase III, randomized trial of mirvetuximab soravtansine versus chemotherapy in patients with platinum-resistant ovarian cancer: primary analysis of FORWARD I. Ann Oncol. 2021;32(6):757-765. 33667670 10.1016/j.annonc.2021.02.017

[49] ImmunoGen, Inc. A study of mirvetuximab soravtansine vs. investigator’s choice of chemotherapy in women with folate receptor (FR) alpha positive advanced epithelial ovarian cancer (EOC), primary peritoneal or fallopian tube cancer (FORWARD I). In: ClinicalTrials.gov. Updated October 14, 2020. Accessed February 10, 2025. https://clinicaltrials.gov/ct2/show/NCT02631876

[50] Coleman RL , Oaknin A, Pignata S, et al Mirvetuximab soravtansine (MIRV) in patients with platinum-resistant ovarian cancer with high folate receptor alpha (FRα) expression: evaluation of sequence of therapy on anti-tumor activity in the SORAYA study. Abstract (19) presented at: Society of Gynecologic Oncology Annual Meeting on Women’s Cancer; March 2023; Tampa, FL.

[51] Moore KN , O’Malley DM, Vergote I, et al Safety and activity findings from a phase 1b escalation study of mirvetuximab soravtansine, a folate receptor alpha (FRα)-targeting antibody-drug conjugate (ADC), in combination with carboplatin in patients with platinum-sensitive ovarian cancer. Gynecol Oncol. 2018;151(1):46-52. 30093227 10.1016/j.ygyno.2018.07.017

[52] Richardson DL , Moore KN, Vergote I, et al Phase 1b study of mirvetuximab soravtansine, a folate receptor alpha (FRα)-targeting antibody-drug conjugate, in combination with carboplatin and bevacizumab in patients with platinum-sensitive ovarian cancer. Gynecol Oncol. 2024;185:186-193. 38447347 10.1016/j.ygyno.2024.01.045

[53] AbbVie. Mirvetuximab soravtansine monotherapy in platinum-sensitive epithelial, peritoneal, and fallopian tube cancers (PICCOLO). In: ClinicalTrials.gov. Updated December 1, 2022. Accessed February 8, 2023. https://clinicaltrials.gov/ct2/show/NCT05041257

[54] AbbVie. Mirvetuximab soravtansine with bevacizumab versus bevacizumab as maintenance in platinum-sensitive ovarian, fallopian tube, or peritoneal cancer (GLORIOSA). In: ClinicalTrials.gov. Updated December 20, 2022. Accessed February 8, 2023. https://clinicaltrials.gov/ct2/show/NCT05445778

[55] AbbVie. Mirvetuximab soravtansine (MIRV) with carboplatin in second-line treatment of folate receptor alpha (FRα) expressing, platinum-sensitive epithelial ovarian cancer. In: ClinicalTrials.gov. Updated November 14, 2022. Accessed February 8, 2023. https://clinicaltrials.gov/ct2/show/NCT05456685

[56] Moore KN , O'Malley DM, Vergote I, et al Mirvetuximab soravtansine and carboplatin for treatment of patients with recurrent folate receptor alpha–positive platinum-sensitive ovarian cancer: a final analysis. Abstract (499) presented at: Annual Global Meeting of the International Gynecologic Cancer Society; September-October 2022; New York, NY.

[57] Stewart M , Rives T, Blanton K, et al Mirvetuximab after anaphylaxis to paclitaxel: a case report. Gynecol Oncol Rep. 2024;54:101452. 39076678 10.1016/j.gore.2024.101452 PMC11284546

[58] Eaton JS , Miller PE, Mannis MJ, et al Ocular adverse events associated with antibody-drug conjugates in human clinical trials. J Ocul Pharmacol Ther. 2015;31(10):589-604. 26539624 10.1089/jop.2015.0064 PMC4677113

[59] Hendershot A , Slabaugh M, Riaz KM, et al Strategies for prevention and management of ocular events occurring with mirvetuximab soravtansine. Gynecol Oncol Rep. 2023;47:101155. 37102083 10.1016/j.gore.2023.101155 PMC10123335

[60] Hesketh PJ , Kris MG, Basch E, et al Antiemetics: ASCO guideline update. J Clin Oncol. 2020;38(24):2782-2797. 32658626 10.1200/JCO.20.01296

[61] Zhu Z , Shen G, Li J, et al Incidence of antibody-drug conjugates-related pneumonitis in patients with solid tumors: a systematic review and meta-analysis. Crit Rev Oncol Hematol. 2023;184:103960. 36907365 10.1016/j.critrevonc.2023.103960

[62] Richardson DL , Eskander RN, O’Malley DM. Advances in ovarian cancer care and unmet treatment needs for patients with platinum resistance: a narrative review. JAMA Oncol. 2023;9(6):851-859. 37079311 10.1001/jamaoncol.2023.0197

[63] Alvarez Secord A , Lewin S, Method M, et al PICCOLO: an open-label, single-arm, phase 2 study of mirvetuximab soravtansine in recurrent platinum-sensitive, high-grade epithelial ovarian cancers with high folate receptor alpha (FRα) expression. Abstract (1556) presented at: Annual Global Meeting of the International Gynecologic Cancer Society; September-October 2022; New York, NY.

[64] AbbVie. A study of ocular toxicity evaluation and mitigation during treatment with mirvetuximab soravtansine in participants with recurrent ovarian cancer with high folate receptor-alpha expression. In: ClinicalTrials.gov. Updated April 15, 2024. Accessed April 16, 2024. https://clinicaltrials.gov/ct2/show/NCT06365853

[65] O’Malley DM , Myers T, Zamagni C, et al GLORIOSA: a randomized, open-label, phase 3 study of mirvetuximab soravtansine with bevacizumab vs. bevacizumab as maintenance in platinum-sensitive ovarian, fallopian tube or primary peritoneal cancer. Abstract (TPS5622) presented at: American Society of Clinical Oncology Annual Meeting; June 2023; Chicago, IL.

[66] O’Malley DM , Myers T, Wimberger P, et al Maintenance with mirvetuximab soravtansine plus bevacizumab vs bevacizumab in FRα-high platinum-sensitive ovarian cancer. Future Oncol. 2024;20(32):2423-2436. 39082675 10.1080/14796694.2024.2372241 PMC11520569

